# Longitudinal Data and Correlated Measures Bias: The Alternative of Mixed Models

**DOI:** 10.36660/abc.20190601

**Published:** 2019-12

**Authors:** Johnnatas Mikael Lopes, Marcello Barbosa O.G. Guedes, Rafael Limeira Cavalcanti, Clecio Gabriel de Souza

**Affiliations:** 1 Universidade Federal do Vale do São Francisco UNIVASF - Colegiado de Medicina, Paulo Afonso, BA - Brazil; 2 Universidade Federal do Rio Grande do Norte UFRN - Fisioterapia, Natal, RN - Brazil; 3 Centro Universitário Maurício de Nassau UNINASSAU - Fisioterapia, Natal, RN - Brazil

**Keywords:** Cohort Studies, Longitudinal Studies, Cross-Section,Studies, Epidemiology, Biostatistics.

Longitudinal studies have two important data typologies: single outcomes or repeated measures.^1^ Single outcome, such as death or disease onset, should have a different data treatment than those studies with repeated measures outcome. But, they have in common the detection of changes over time and the contributing factors for this change. Cohort differs from cross-sectional studies that desire only variables relationshiP , without causal effect.

Fernandes et al.^2^ wrote an article entitled *The RelationshiP between Lifestyle and Costs Related to Medicine Use in Adults*, published in this journal, volume 112, number 6, 2019, and they used behavioral independent variables to estimate their effects on drug costs outcome, collected as repeated measurements in a prospective cohort design.

The aim of this exposition is to show that, probably, there was a mistake in the Fernandes et al.^2^ data analysis, which compromises the causality inferences due to the great possibility of the estimates’ accuracy to be mistaken.

Let's get to the facts. Considering the prospective cohort design with repeated measures, there is a hierarchical structure in the outcome data due to their clustering in the same participant after various measures. Data cluster leave to the model error, that is the difference between what was predicted by the model and the actual measurement, of the same participant, at different times, to be correlated.^3^ This is a condition for not using multiple linear regression (MLR) which assumes the independence of the model error given by the assumption that the distribution of each participant is equal. MLR does not extract from the data which is variability within the individual from variability between individuals (population).^3^

Using RLM in repeated measures generates regression coefficients with standard errors biased. This requires covariance matrix application that will produce more reliable estimates, in others words, narrower confidence intervals from Mixed Effects Models.^4^ This is the best alternative to verify changes over time or the conditioners effects on repeated measures outcomes in longitudinal studies, controlling for individual effects.

There is greater variability between individuals than within individuals, mainly due to biological and social conditioning differences, it’s observed that drug costs will be more correlated over time in the same individual than among participants. To think that this distribution is the same among the participants ignores theoretical assumption in the social determination on people's behavior.^5^

Build distinct MLRs (A, B, C and D), see Fernandes et al.^2^, does not control this covariance effect, and therefore may be producing coefficients with confidence intervals biased in independent variables and can not detect the rate of change from basal either.^3^ In addition, with mixed models it would also be possible to take advantage of measurements that were measured on lost participants, increasing modeling sensitivity.^4^

From another perspective, the objective of the research being to estimate the interrelation of drug cost and behavioral habits, without establishing causality, would only require a cross-sectional design of the participants with the collection of outcome data and independent variables at a single moment. Thus, the basal regression model would be sufficient to estimate gross and adjusted associations.^1^

Thus, the use of RLM should be restricted to cross-sectional research designs and longitudinal studies with repeated measures outcomes need to differentiate the individual effect of the population effect in the identification of temporal changes and their conditioning. Possibly, the findings of Fernandes el al.^2^ should be based regarding their conclusions about the inverse relationshiP between alcohol use and drug costs or the statistically non-significant relationships with body fat, gender and smoking status that have great impact on other health situations, especially chronic diseases.
