# Supplementary material for: Longitudinal Data and Correlated Measures Bias: The Alternative of Mixed Models
Source: Arq Bras Cardiol. 2019 Dec;113(6):1155–7. doi: 10.36660/abc.20190601 (PMC7021255; doi:10.36660/abc.20190601)
Supplement: Related texts

# Reply

**Authors:** Rômulo Araújo Fernandes

I appreciate the opportunity to answer the questions concerning our manuscript recently published in Arquivos Brasileiros de Cardiologia.^1^ Academic discussion is always healthy and welcome.

Firstly, thank you for your interest in our study. The question raised refers to the use of linear regression in the treatment of data from a prospective cohort with repeated measures, which is believed to have caused mistaken estimates (mixed linear regression is suggested instead). Linear regression is debated as it fails to detect intra-individual variability properly, as it focuses on variability between individuals. From a theoretical point of view, this statement is correct, but it does not reflect the way the data were analyzed in the study.

The dependent variable of the study was defined as “drug spending over 12 months.” In the study, we did not analyze the history of drug spending over the year^2^ (and how this history would be affected by behavioral variables), nor did we seek to identify the relationshiP between changes compared to baseline (for dependent and independent variables). We did try to analyze the relationshiP of behavioral variables with the final amount spent over the year.

In fact, this dependent variable is unusual in its construction, as it was longitudinally designed (expenditures on drugs computed over 12 months), but treated cross-sectionally (total amount spent over 12 months). The total amount of drug spending reflects a cross-sectional construct, although its construction considers the 12 months of follow-uP . This particularity of the dependent variable, added to the fact that the behavioral variables were collected at only two moments (baseline and at the end of 12 months), led us to create the four models proposed in the study, which characterize a cross-sectional view of the problem (especially models A [baseline data] and B [at the end of 12 months]). Unfortunately, the monthly assessment of behavioral variables was not an available methodological option.

In an ideal model, the dependent variable and the independent variables should be collected monthly, allowing to identify the impact of changes on behavioral variables on changes in drug spending history over the year. However, I repeat, this was not the purpose of the study.^1^ For this type of analysis, specific structural equation modelling (latent growth curve analysis) would be more suitable (even more so than mixed linear regression), as they would make it possible to analyze the direct impact of changes on independent variable (slope)over changes observed on dependent variable (slope).^3^ The “impact” measures generated by the model are easily interpreted, as they can be expressed as correlation coefficients, which additionally provide effect-size measurements.^4^

Additionally, the dependent variable as it was presented (cross-sectionally, with spending accruing over follow-uP time) was necessary due to the particularities observed in its structure. Unlike other variables usually measured in different areas of health sciences (height, blood pressure, lipid profile components), which do not have zero value, drug spending occurs irregularly, reflecting the high occurrence of zero values (that is, spending can be reported in the first month of collection, then no spending can be reported over the subsequent months). Against this background, analyses considering the month-to-month variable would be problematic. Likewise, the issue of intra-individual variability needs to be considered with caution in this study because drug spending in the previous month does not recur in the following month, unlike what was observed for variables like height^5^ which, even without any gain, the amount of the previous month will repeat in the following month.

Finally, the absence of significant relationships for obesity and smoking is not surprising in this study, as the sample is relatively young, without the presence of chronic diseases and low occurrence of smoking.

## References

[1] Fernandes RA, Mantovani AM, Sanches Codogno J, Camilo Turi-Lynch B, Pokhrel S, Anokye N (2018). Relação entre Estilo de Vida e Custos Relacionados ao Uso de Medicamentos em Adultos. Arq Bras Cardiol.

[2] Lemes ÍR, Fernandes RA, Turi-Lynch BC, Codogno JS, de Morais LC, Koyama KAK, Monteiro HL (2019). Metabolic Syndrome, Physical Activity, and Medication-Related Expenditures: A Longitudinal Analysis. J Phys Act Health.

[3] Brailean A, Aartsen MJ, Muniz-Terrera G, Prince M, Prina AM, Comijs HC, Huisman M, Beekman A (2017). Longitudinal associations between late-life depression dimensions and cognitive functioning: a cross-domain latente growth curve analysis. Psychol Med.

[4] Maher JM, Markey JC, Ebert-May D (2013). The other half of the story: effect size analysis in quantitative research. CBE Life Sci Educ.

[5] Fausto MA, Carneiro M, Antunes CM, Pinto JA, Colosimo EA (2008). Mixed linear regression model for longitudinal data: application to an unbalanced anthropometric data set. Cad Saude Publica.
